# Is cardiac surgery training really hazardous?

**DOI:** 10.1186/1749-8090-8-S1-O94

**Published:** 2013-09-11

**Authors:** M El Saegh, H El Saegh, T Theologou, P Yiu

**Affiliations:** 1 Department of Cardiothoracic Surgery, The James Cook University Hospital, Middlesbrough, UK; 2 Department of Cardiology, The National Heart Institute, Imbab, Giza; 3 Department of Cardiothoracic Surgery, New Cross Hospital, Wolverhampton, UK

## Background

Most of the times patients, and sometimes surgeons (even trainees) may feel that cardiac surgery training isn’t very safe, and may affect the outcome.

## Methods

1 year retrospective post-operative analysis of 520 patients who underwent isolated CABG surgery based on morbidity and mortality. The patients were divided into: patients operated by consultants (C), 444 patients and patients operated upon by registrars under supervision (F); 76 patients.

## Results

Our results showed highly significant difference in the registrars group compared to consultants for cumulative cardiopulmonary bypass time, and aortic cross clamp time; p <0.01. While there was significant difference concerning post operative chest infection, p<0.05 in the registrars group. There was no statistically significant difference in logistic euro score, in-hospital mortality, incidence of post operative infection, post operative renal impairment, post operative arrhythmias, ICU readmission, post operative stay, reopening or the need for inotropes.

## Conclusion

Cardiac surgery training isn’t really a compromise on outcome of patients undergoing CABG.

